# Insights on associations between the frequency of use of diverse social media products and social networks use disorder tendencies from a German speaking sample

**DOI:** 10.1186/s13104-024-06833-5

**Published:** 2024-07-05

**Authors:** Christian Montag, Elisa Wegmann, Lasse David Schmidt, Lena Klein, Dmitri Rozgonjuk, Hans-Jürgen Rumpf

**Affiliations:** 1 Department of Molecular Psychology, Institute of Psychology and Education, Ulm University, Helmholtzstr. 8/1, 89081 Ulm, Germany; 2 General Psychology: Cognition and Center for Behavioral Addiction Research (CeBAR), Faculty of Computer Science, University of Duisburg-Essen, Duisburg, Germany; 3 Department of Psychiatry and Psychotherapy, University of Lübeck, Lübeck, Germany

**Keywords:** Social media addiction, TikTok, Facebook, Instagram, WhatsApp

## Abstract

**Objective:**

In the present work we investigate how individual differences in at least occasionally using distinct social media platforms is linked to social networks use disorder (SNUD) tendencies. A final sample of *n* = 2200 participants filled in the AICA-C-9 measure to get insights into individual differences in overuse of social media and participants also indicated which platforms they used at least once a month.

**Results:**

The analysis revealed a robust positive association between number of at least occasionally used social media apps and SNUD tendencies (*r* = .44, *p* < .001). Further, platforms differed in terms of their “addictive potential”, if one takes associations between frequency of distinct platforms use and SNUD tendencies as a proxy for this (and of course the actual descriptive statistics of the SNUD scale for the (non-)frequent user groups of the different platforms). In this regard, at least occasionally using some platforms (here Tumblr, Twitter and TikTok) was associated with highest SNUD tendencies. Moreover, largest differences in terms of effect sizes between the occasional and non-occasional user groups regarding SNUD scores could be observed for Instagram, WhatsApp, and TikTok. The present work bases on data from a larger project investigating associations between SNUD and tobacco use disorder.

## Introduction

According to current estimates nearly five billion people are using a social media service worldwide [1] representing a communication tool differing in context from classic face-to-face or digital communication from such as via e-mail [2] or videoconferences [3]. Differences of context in social media platforms and offline lives might also result in differences in the perception of one’s own person [4]. Most of the current operating social media platforms run with a data business model, hence users do not pay with a monetary fee to use the service, but instead with their attention to ads and with their data [5]. In turn, the companies behind the social media services can create profiles of their users to improve their business case around personalized ads [6]. For years, it has been discussed if people can get “addicted” to social media services [7]. Although this debate is not settled [8], one could take the position that an industry actively aiming at prolonging online time represents one of the causes of excessive social media use [9, 10]. Although social media addiction or social networks use disorder (SNUD) does not represent an official diagnosis now, scientists around the world often use an addiction framework to study overuse of social media [11]. In this context, they assess disordered social media use by asking people about the severity of symptoms which have been inspired by addiction research [12]. Among these are loss of control, withdrawal, preoccupations, etc. Please note that recent research demonstrated that only a few of such addiction symptoms are associated with psychopathological tendencies [13]. In addition, everyday use of digital media or devices should not be overpathologized as, for example, lined out in relation to gaming disorder [14]. To better distinguish between everyday behavior and pathology, theoretical underpinnings [15] and nosological criteria are of high importance. Most often, diagnostic criteria of Internet Gaming Disorder in the Diagnostic and Statistical Manual of Mental Disorders in its 5th revision (DSM-5; [16, 17]) or diagnostic requirements of Gaming Disorder in the 11th revision of the International Classification of Diseases (ICD-11; World Health Organization)^1^; see also [18] have been adapted for this purpose.

Prominent scales used in the field to assess disordered social media use often assess general social media overuse without a focus on distinct platforms [12, 19]. This said, recent research suggests that some platforms might elicit more disordered use tendencies than others [20], and might have a stronger impact on daily-life adversities [21]. One explanation for this is that platforms differ in their design and could therefore exert a different pull to come to the platform [22]. Studying several social media platforms in a single work might help to overcome biases in findings from studies focusing on single prominent large networks such as Facebook [23]. Therefore, the present research asks study participants about their disordered general social networks use tendencies *and* in detail which of a longer list of social media platforms they use at least on a monthly level. This information is brought together to see if use of certain platforms goes along with elevated SNUD tendencies. We aim to investigate which use of social media services is related with highest SNUD tendencies to provide further insights into the potential addictive features of different platforms. Moreover, we analyze if more social media services are used by one person is related to higher SNUD tendencies as well.

## Methods

### Participants, data cleaning and questionnaire measures

An initial sample of *N* = 2219 participants could be recruited via a project website representing the starting point for investigating associations between social media use and smoking (data of a small subsample of the present sample (about ¼) regarding the AICA-C-9 has been investigated in the context of smoking before [24]). The detailed methods and agenda of the overarching project(s) have been presented in recent works [25, 26].

Four participants were under 16 years and therefore were excluded, because the protocol foresaw 16 years and older as entry age for the present study. Further two participants were excluded due to providing implausible age information. Thirteen participants were also excluded from the analysis being characterized as third gender. This group is unfortunately too small to run robust statistics on (the investigation of this group represents an important research topic though from our view).

The final sample consisted of *N* = 2200 participants with *n* = 1454 females (66,1%) and *n* = 746 males (33,9%). All participants provided insights into their smoking behavior (smoker (33,1%), ex-smoker (14,3%) and non-smoker (52,5%- the smoking variables are not of relevance for the present project; again see analysis in a smaller subsample here [24])^2^ and into their social media use disorder tendencies and what platforms they used at least occasionally. Therefore, we presented several messengers and platforms (Facebook and Facebook Messenger, Instagram, iMessage, Telegram, WhatsApp, Threema, Signal, Snapchat, TikTok, Twitter, Tumblr, Pinterest, Skype, other category). As one can see both social media and messenger platforms are mentioned in the above list, because messengers such as WhatsApp have social media features such as online status and according to a visible social media definition messengers would belong under the umbrella term social media [27]. A “yes/no” variable (at least occasionally/less than once a month use) for each platform was used. Usage of each platform at least *once a month* was indicated with a yes = 1 or *less frequently* with a no = 0. Further a variable was computed (a summed score of all at least occasionally used social media platforms) to understand how many different social media products the study participants used frequently. A note: We have five participants (0,2%), who not reported to use any of the mentioned platforms recently. Nevertheless, it could be that they very seldom use these platforms. We did not exclude them from the analysis (for sure this group is very small and will not have a large impact).

Individual differences in SNUD tendencies were assessed via the AICA-C-9 questionnaire (the original AICA-S scale with English wording can be found here [28]; please note that in this original paper the wording gaming was used, and in the present work the focus was on social networks). The shortened AICA-C-9 questionnaire consists of twelve items (one presented in an adaptive way), which can be used to assess nine symptom criteria of SNUD developed against the Internet Gaming Disorder framework in DSM-5 [29]. Higher scores indicate higher SNUD tendencies. Cronbach’s α for the nine symptoms was 0.76 and therefore satisfying. Finally, all participants provided insights into what operation system they used on their phones (*n* = 1052 used iOS (47,8%), *n* = 1137 used Android (51,7%) and *n* = 11 (0,5%) used other operation systems.

### Statistical analyses

**Fig. 1:**
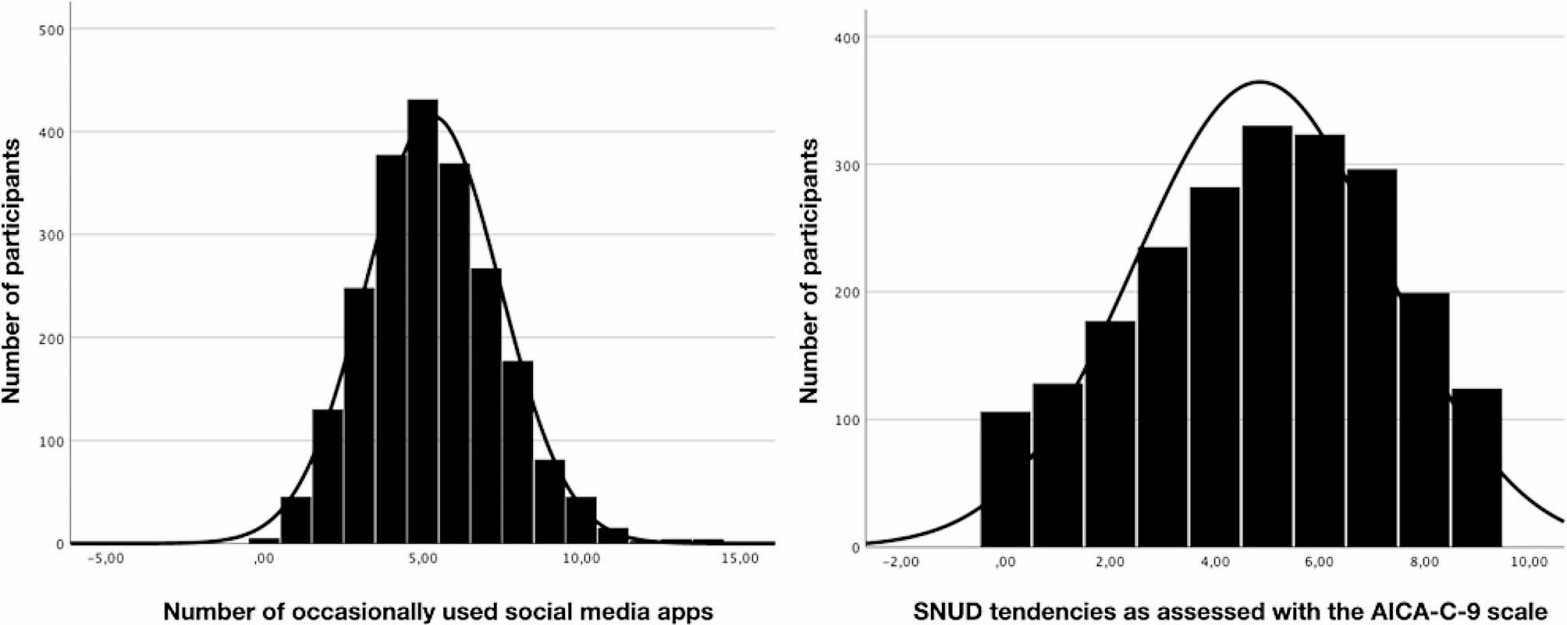
Distribution of at least occasionally used social media apps and AICA-C-9 scores (SNUD tendencies)

As one can see the key variables AICA-C-9 and the number of social media products used at least occasionally are rather normally distributed and therefore parametric tests were applied (also due to the fact that the sample is very large; see Fig. 1).

A Pearson correlation between AICA-C-9 and number of social media products used provide insights into a potential association between both variables. Additionally, individual t-tests are presented showing how (not) at least occasionally use of/using each platform is linked to the AICA-C-9 scores (contrast between occasional and non-occasional users of distinct social media platforms). As exploratory analysis, also associations between the operation system variable and social media use was investigated. For the analysis SPSS 29.0.1.0 and Jamovi 2.4.8.0 were used. As social media overuse - according to some studies - is related to age and gender, we also checked if these variables need to be controlled for [7]. The data underlying this work can be found here: https://osf.io/9j8g3/.

## Results

### Age, gender and SNUD tendencies/number of social media apps used

Analysis suggests that significant differences between men and women regarding SNUD tendencies (AICA-C-9 scores) could be observed (but effect size is weak). Women had slightly higher scores than men (*t*_(1368,102)_ = 2.107, *p* = .035; women: *M* = 4.94 (*SD* = 2.31) vs. men: *M* = 4.70 (*SD* = 2.58); Cohen’s d = 0.098). Women and men did not significantly differ regarding the number of at least occasionally use of apps with women having higher scores than men (*t*_(1329,923)_ = 1.18, *p* = .264; females: *M* = 5.35 (*SD* = 2.01) vs. males: *M* = 5.24 (*SD* = 2.31)). Against this background we did not control for gender in the following analysis (the readers can use the data for further analysis, if they are interested, because they are shared open access). Age was robustly and negatively associated with AICA-C-9 scores (*r* = − .241, *p* < .001) and with number of at least occasionally social media apps used (*r* = − .225, *p* <. 001). Hence younger age was associated with more SNUD tendencies and with a higher number of at least occasionally used social media apps. Therefore, we also ran a partial correlation between AICA-C-9 scores and number of social media apps at least occasionally used controlling for age. Correlations are presented on two-sided level.

### Further insights into social media use

Descriptive statistics revealed how many percent of the users stated to use a certain social media product at least occasionally (at least once a month; see Table 1). The most often used app was WhatsApp (97%), the least often used apps were Threema/Tumblr (5%).

**Table 1:** Number of participants in % stating to use the presented platform at least once a month

Name of the platforms	Number of participants in % stating to use the platform at least once a month (*n* in brackets)
Facebook and Facebook Messenger	55,1% (1213)
Instagram	80,4% (1769)
IMessage	26,0% (572)
Telegram	27,6% (607)
WhatsApp	97,0% (2135)
Threema	5,0% (109)
Signal	26,6% (586)
Snapchat	42,0% (925)
TikTok	43,9% (966)
Twitter	21,9% (482)
Tumblr	5,0% (109)
Pinterest	43,5% (957)
Skype	16,2% (356)
Others	41,0% (901)

**Fig. 2:**
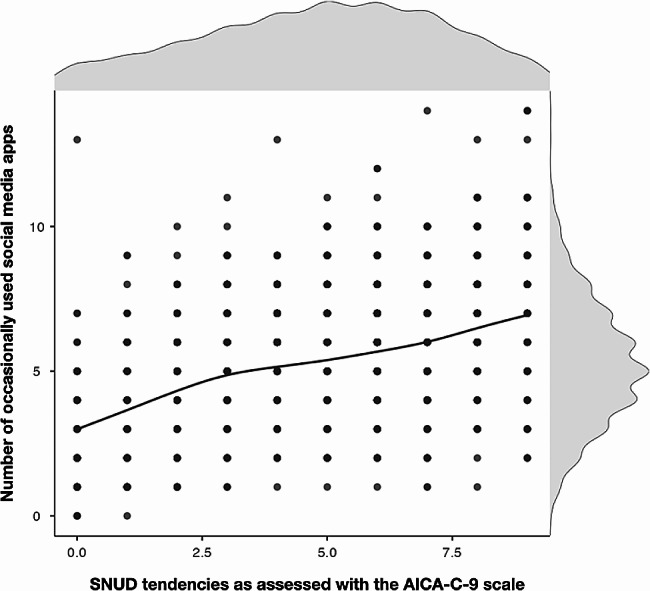
Scatterplot illustrating the associations between number of frequently used social media apps and SNUD tendencies

On average, participants reported to use a mean of 5.31 apps at least occasionally (*SD* = 2.12) and a mean AICA-C-9 score of 4.86 (*SD* = 2.41). A moderate association could be observed between number of at least occasionally social media apps used and the AICA-C-9 scores: *r* = .44, *p* < .001 (see for illustration Fig. 2). Controlling for age led to a slight reduction of the observed effect size: *r* = .41, *p* < .001.

Interestingly, we observed that for all platforms at least occasionally used was associated with descriptively higher SNUD tendencies (AICA-C-9 scores) compared to less-frequent use, except for Threema, where we observed the contrary patterns (although not significant); see Table 2. Therefore, we created also a sum score for number of frequently used social media apps without Threema. Correlations did not really change then (*r* = .45, *p* < .001; controlling for age *r* = .41, *p* < .001).

**Table 2:** The descriptive statistics are presented for the AICA-C-9 scores depending on at least occasionally use vs. less-frequent use

Name of the platforms	At least occasionally use	Less-frequent use	T-Test and effect size
Facebook and Facebook Messenger	M = 5.09 (SD = 2.39)	M = 4.57 (SD = 2.40)	t_(2198)_ = 5,099, *p* < .001, Cohen’s d = 0.219
Instagram	M = 5.26 (SD = 2.18)	M = 3.19 (SD = 2.60)	t_(585,382)_ = 15,257, *p* < .001, Cohen’s d = 0.912
IMessage	M = 5.45 (SD = 2.38)	M = 4.65 (SD = 2.38)	t_(2198)_ = 6,924, *p* < .001, Cohen’s d = 0.337
Telegram	M = 5.38 (SD = 2.41)	M = 4.66 (SD = 2.38)	t_(2198)_ = 6,304, *p* < .001, Cohen’s d = 0.301
WhatsApp	M = 4.91 (SD = 2.38)	M = 3.11 (SD = 2.74)	t_(66,969)_ = 5,242, *p* < .001, Cohen’s d = 0.754
Threema	M = 4.56 (SD = 2.39)	M = 4.87 (SD = 2.41)	t_(2198)_ = 1,318, *p* = .188, Cohen’s d = − 0.129
Signal	M = 4.86 (SD = 2.48)	M = 4.85 (SD = 2.38)	t_(2198)_ = 0.049, *p* = .961, Cohen’s d = 0.002
Snapchat	M = 5.48 (SD = 2.10)	M = 4.41 (SD = 2.52)	t_(2155,550)_ = 10,862, *p* < .001, Cohen’s d = 0.456
TikTok	M = 5.66 (SD = 2.13)	M = 4.22 (SD = 2.43)	t_(2170,555)_ = 14,831, *p* < .001, Cohen’s d = 0.627
Twitter	M = 5.75 (SD = 2.25)	M = 4,60 (SD = 2,39)	t_(811,910)_ = 9,750, *p* < .001, Cohen’s d = 0.485
Tumblr	M = 5.78 (SD = 2.29)	M = 4,81 (SD = 2,40)	t_(2198)_ = 4,124, *p* < .001, Cohen’s d = 0.405
Pinterest	M = 5.43 (SD = 2.16)	M = 4.42 (SD = 2.49)	t_(2166,500)_ = 10,168, *p* < .001, Cohen’s d = 0.429
Skype	M = 5.30 (SD = 2.43)	M = 4,77 (SD = 2.40)	t_(2198)_ = 3,818, *p* < .001, Cohen’s d = 0.221
Others	M = 5.49 (SD = 2.25)	M = 4.42 (SD = 2.42)	t_(2023,705)_ = 10,656, *p* < .001, Cohen’s d = 0.456

On a descriptive level, highest SNUD tendencies could be observed in those reporting to at least occasionally use of Tumblr, Twitter, and TikTok. Largest differences in terms of effect sizes between the groups of at least occasionally and less-frequent use of the different platforms could be observed for Instagram, WhatsApp, and TikTok.

### Exploratory analysis: operation system and social media use

ANOVA revealed that the operation system was associated both with the AICA-C-9 scores (F_(2,2197)_ = 28,64, *p* < .001, eta^2^ = 0.025) and the number of at least occasionally used social media apps (F_(2,2197)_ = 46,08, *p* < .001, eta^2^ = 0.040). iOS users reported higher SNUD tendencies (M = 5.26, SD = 2.32) than Android users (M = 4.49, SD = 2.43) followed with lowest scores in the others group (M = 4.36, SD = 2.16). Fittingly, iOS users reported also the highest number of at least occasionally used apps (M = 5.75, SD = 2.11) followed by Android users (M = 4.93, SD = 2.04) followed with number of at least occasionally used apps in the others group (M = 3.64, SD = 1.75).

## Discussion

The present work aimed to give detailed insights into associations between frequent (at least occasionally) use of different social media apps and self-reported SNUD tendencies.

First, we observed that a larger number of at least occasionally used social media apps correlated positively and moderately with higher SNUD tendencies. A reason for this might be that people prone to overusing social media either might prefer the consumption of different platforms (offering for instance videos such as on TikTok vs. more still pictures such as on Instagram). On the other hand, using more platforms could also elicit a stronger pull towards social media in general, which can also lead to mechanisms that lead to continuous consumption, such as FoMO, being further reinforced across the various platforms [22, 30, 31].

Second, we observed that at least occasionally use of some platforms was in particular associated with higher SNUD tendencies (highest scores for Tumblr, Twitter and TikTok), which fits with the idea that not all social media products are identical and that differences in the design of the apps could elicit different addictive tendencies towards the platform [20, 22]. In this context it should be mentioned that different platforms in the beginning of usage might elicit different habit formation giving way to addictive tendencies towards distinct social media platforms [32]. A recent work further suggests “that while the underlying addiction pathways are similar between platforms the manifestation of maladaptive behavior and the drivers for usage intensity and problematic use are unique.“ [33].

Largest differences in terms of effect sizes between the groups could be observed for Instagram, WhatsApp, and TikTok. At this point, it is merely speculative as to why certain platforms may be more attractive or associated with a different addictive potential than others, future studies will need to examine the role played by certain design features, familiarity, algorithms and their speed of adaptation, as well as the content itself of the different platforms [22, 34, 35]. Furthermore different platforms attract different user groups [36], which could also explain differences in SNUD tendencies towards certain social media applications. To illustrate this: As a further analysis for the discussion, we investigated age differences between frequent and non-frequent TikTok users. At least occasionally users were much younger than less-frequent users (M = 28.30 (SD = 10.51) vs. M = 33.37 (SD = 12.53) and it is also known that younger age goes along with higher SNUD tendencies [7], what we also observed in the present work. If readers are interested in further investigating age and gender variables in the context of certain platform use, the data is available for further analysis (OSF: https://osf.io/9j8g3/).

### Limitations

The present study comes with several limitations. First, the present study is a self-report study and therefore can be biased by lack of introspection and answering in a socially desirable fashion. Second, the present study is of cross-sectional nature and consequently no causality can be derived. Third, the assessment of frequency of social media use should be more fine-granular in the future and should go beyond usage of once a month vs. more seldom. Fourth, the present sample is a convenience sample, hence the generalization of results may be limited (might be also biased due to the recruiting strategy inviting participants for a larger study on smoking and social media use). Finally, some of the investigated social media groups in this work are small and statistics might not be robust here (e.g. only 5% mentioned to use Tumblr and Threema at least occasionally).

## Conclusion

In conclusion, the present work underlines the idea that not all social media platforms are the same in terms of their addictive potential and that distinct analysis of platforms are required. Beyond this, clearly more use of social media apps goes along with higher addictive tendencies towards social media in general. Findings might be used for considering convenient assessment of simple markers to detect individuals at-risk for addictive use of social media. Frequency of use and the total number of social media apps used could serve as early signs in digital phenotyping or mobile sensing [37]. Moreover, they might be used in addition to or as a part of screening questionnaires. Early detection is a prerequisite for intervention approaches in terms of indicative prevention as stand-alone measure or within a comprehensive stepped-care approach [38].

## Data Availability

The data is available at the Open Science Framework: https://osf.io/9j8g3/).

## Footnotes

1 https://icd.who.int/browse/2024-01/mms/en#/http://id.who.int/icd/entity/1448597234.

2 In this much larger sample, we could again observe that smokers were associated with lowest AICA-C-9 scores (here: 4,62 (SD = 2,55) compared to non-smokers (M = 4,91 (SD = 2.31)) and ex-smokers (M = 5,19 (SD = 2,38)). ANCOVA revealed a significant effect (F_(2,2197)_ = 6,858, *p* = .001).
